# Use of a LiESP/QA-21 Vaccine (CaniLeish) Stimulates an Appropriate Th1-Dominated Cell-Mediated Immune Response in Dogs

**DOI:** 10.1371/journal.pntd.0001683

**Published:** 2012-06-19

**Authors:** Javier Moreno, Ioannis Vouldoukis, Virginie Martin, David McGahie, Anne-Marie Cuisinier, Sylvie Gueguen

**Affiliations:** 1 WHO Collaborating Centre for Leishmaniasis, Centro Nacional de Microbiologia, Instituto de Carlos III, Madrid, Spain; 2 INSERM UMR S 945, Immunité et Infection, Faculté de Médecine Pitié-Salpêtrière, Université Pierre et Marie Curie, Paris, France; 3 Biological R&D, Virbac, Carros, France; 4 Medical Department, Virbac, Carros, France; Queensland Institute of Medical Research, Australia

## Abstract

Canine leishmaniasis is an important zoonotic disease of dogs. The clinical outcome of infection is variable, with the efficiency of the immune response being the key determining factor. There is now a general consensus that a predominant Th1 immune profile in an overall mixed Th1/Th2 response is associated with resistance in dogs, and the absence of a strong Th1 influence is associated with a progression to clinical disease. As a result, there has been a growing demand for vaccines that can induce a specific, strong Th1 response. In this study, we measured the impact of a primary course of a newly available LiESP/QA-21 vaccine on selected humoral and cellular markers of the canine immune response during the onset of immunity. All vaccinated dogs developed a humoral response characterised by IgG2 production. More importantly, vaccinated dogs developed significantly stronger cell-mediated immunity responses than did control dogs. Vaccination induced specific cellular reactivity to soluble *Leishmania* antigens, with a *Leishmania*-specific lymphoproliferation (p = 0.0072), characterised by an increased population of T lymphocytes producing IFN-γ (p = 0.0021) and a significant ability of macrophages to reduce intracellular parasite burdens *in vitro* after co-culture with autologous lymphocytes (p = 0.0014). These responses were correlated with induction of the NOS pathway and production of NO derivatives, which has been shown to be an important leishmanicidal mechanism. These results confirm that vaccination with LiESP/QA-21 induces an appropriate Th1-profile cell-mediated response within three weeks of completing the primary course, and that this response effectively reduces the parasite load in pre-infected macrophages *in vitro*.

## Author Summary

The dog is the principle reservoir of *Leishmania infantum*, a parasite spread from dog to dog by a sandfly vector. The reduction of canine leishmaniasis is therefore a key factor in the overall management of the epidemiology of this parasite. There is also a need for effective prevention on welfare grounds because of the clinical severity of this potentially fatal disease in dogs. The key factor determining the outcome of infection in dogs is the ability to mount a Th1-dominated immune response, because this is more effective against intracellular pathogens such as *L. infantum*. Until now, in Europe, only measures to reduce sandfly bites have been available, and for many years there has been a strong demand for a vaccine that provides specific and effective immunity. The recent launch of the first European canine leishmaniasis vaccine (CaniLeish) has provided a means to achieve this, but data on its impact on the dog's immune system are required. The data presented here demonstrate the specific stimulation of an effective Th1-dominated anti-*L. infantum* response within three weeks of the administration of the vaccine, and provides a basis for the understanding of the mode of action of this new tool.

## Introduction

Canine leishmaniasis, a vector-borne disease of dogs, is caused by *Leishmania infantum* in the Mediterranean basin and is a significant problem for the canine population of endemic areas [1]. It is transmitted in the Mediterranean area by the bite of certain species of sand flies of the *Phlebotomus* (*Larroussius*) subgenus, and so natural transmission can only occur in areas where competent vector species are present [2]. Currently it is estimated that at least 2.5 million dogs are infected in southwestern Europe alone [3] and recent publications have reported a northward spread of the endemic area [4]. Given that canine leishmaniasis is a potentially severe and fatal disease, it represents a source of suffering for affected dogs and many dog owners are highly concerned about how best they can protect their animals. Moreover, the dog is the principle reservoir for human infection; thus a high prevalence of this canine disease also represents a zoonotic risk [5].

In recent years, several topical repellent and insecticide preparations with good trial data have become available and these are able to decrease the intensity of parasite challenge received by the dog by decreasing the number of infectious sandfly bites received. However although these products have good short-term efficacy data when used on an individual dog basis, some evidence exists to suggest that this may not be maintained over the longer term [6]. Even with correct use, these products cannot prevent all infectious bites and there is still a need for further control measures [7].

The outcome of the infection in individual dogs is highly variable and not all dogs which are infected will develop the disease [8]. Some dogs will completely clear the infection, some will remain subclinically infected, while some will develop clinical disease of varying severity from mild papular skin disease to severe generalised disease characterised by renal failure and eventually death [9], [10].

Studies on canine leishmaniasis have somewhat clarified the risk factors determining the likelihood of disease developing after infection [1], [3]. However it has become clear that the final outcome of infection depends mainly on the efficiency of the dog's immune system [3], [11]. In murine cutaneous leishmaniasis, it has been demonstrated that there is a clear dichotomous immune response: T helper 1 (Th1) responses are associated with protection while T helper 2 (Th2) responses are associated with the development of disease [12]. In contrast, data from human visceral leishmaniasis cases and the murine model of visceral leishmaniasis have shown that this Th1/Th2 dichotomy is lacking, and a mixed response is essential for protection [13]–[15]. Although dogs which remain asymptomatic after infection with *L. chagasi* ( = *L. infantum*) develop a predominantly Th1 profile response, whereas oligosymptomatic and symptomatic dogs present a Th2 profile [16], a clear dichotomous Th1/Th2 pattern is also lacking in this species [17]. However, despite the fact that the situation is extremely complex, it is now widely accepted that the protective canine immune response is mediated by a dominant CD4+ Th1 influence in an overall mixed cellular response [18], [19]. This protective response is believed to be mediated by the induction of inducible nitric oxide synthase (iNOS) in macrophages upon stimulation by Th1 cytokines such as interferon gamma (IFN-γ). This results in a decreased production of arginase, and consequently of the polyamides that are essential for parasite growth and survival in the macrophage, and an increased production of leishmanicidal nitric oxide (NO) derivatives [20], [21]. In addition, *in vitro* studies propose that while iNOS activity can be considered as an essential effector mechanism to prevent multiplication of *Leishmania* amastigotes, the NO derivative produced may have additional roles including immunoregulatory functions [22].

Because of this pivotal role for the immune system, several authors have expressed the opinion that an effective vaccine against canine leishmaniasis would be the best control strategy for both canine and human disease [7], [23].

Two canine vaccines have been available for some time now in Brazil [11]. However, until the recent launch of the LiESP/QA-21 vaccine (CaniLeish, Virbac, France), there were no vaccines against *Leishmania* available in Europe. With any new vaccine, and especially one that is the first of its kind, it is important to understand as much as possible about the mechanism of its action on the dog's immune response and to study known markers of resistance to disease. Indeed investigation of such parameters has recently been proposed as representing an important supplementary data set when assessing any candidate vaccine for canine leishmaniasis [11]. The aim of the study presented here was to follow selected humoral and cellular markers of the immune response in dogs vaccinated with LiESP/QA-21 vaccine during the establishment of the immune response, and specifically to assess if an effective Th1-dominated profile could be generated.

## Materials and Methods

### Ethics Statement

The Virbac Ethical Committee approval confirms that this study was carried out in accordance with the G.R.I.C.E. “Ethical Committee Regulation applied to animal experimentation” guidelines (implemented in France in 2007).

### Animals' Characteristics

20 conventional Beagle dogs (10 male and 10 female) aged 6 months +/−1week on the day of the first vaccination were randomly assigned to two groups (vaccinated and control) according to their weight, sex and litter of birth. There were 5 males and 5 females per group.

All animals were previously vaccinated with conventional vaccinations against Distemper virus, Adenovirus, Parvovirus, Parainfluenza virus and Leptospira.

They were housed in controlled conditions, and dewormed with nitroscanate (Troscan, Virbac, France) 1 week prior to the date of the first administration of the LiESP/QA-21 vaccine.

### Vaccine and Vaccination Protocol

The LiESP/QA-21 vaccine is authorised in the European Union under the trade name CaniLeish (Virbac, France). It is composed of purified excreted-secreted proteins of *Leishmania infantum* (LiESP), produced by means of a patented cell-free, serum-free culture system invented by the IRD (Institut de Recherche pour le Développement) [24], and adjuvanted with QA-21, a highly purified fraction of the *Quilaja saponaria* saponin. The doses used in this study were formulated at 100 µgESP and 60 µg QA-21. This is consistent with the minimum accepted levels in commercially available doses.

Dogs in the vaccinated group were given one dose of the LiESP/QA-21 vaccine every 21 days for a total of three doses. Dogs in the control group did not receive any vaccination.

### Analyses and Schedule

#### Serology testing of the humoral immune response

ELISA testing was performed on the day of each vaccination (D0, D21, D42) and also two weeks after the last vaccine (D56) to dose the level of IgG1 and IgG2 antibodies to both LiESP and also specifically to Parasite Surface Antigen (PSA), which is a major antigenic component of LiESP. Blood was collected in uncoated tubes and the serum separated before performing the analyses.

Briefly, the technique is performed as follows. A NUNC Maxisorp plate is coated with either 0.1 µg ESP or 0.1 µg PSA per well in carbonate buffer for 90 minutes at 35–37°C. Non-specific sites are blocked with PBS-Tween 0.5%-milk 5% for 90 minutes at 35–37°C. Then serial three-fold dilutions of the serum to be tested in PBS-milk 0.5% buffer, beginning at 1/150, are added to the plate. After 60 minutes of incubation at 35–37°C any antibodies fixed to the ESP or PSA respectively are revealed with a specific peroxydase-conjugated polyclonal anti-IgG1 or anti-IgG2 secondary antibody (Bethyl Laboratories, Montgomery, USA) and ABTS colouration. The titre corresponds to the first dilution with an optical density at 405 nm inferior to 0.4.

#### Cellular immune response assays

The three cell-mediated immunity tests, as described below, [Lymphoblastic Transformation Test (LTT), IFN-γ Enzyme-Linked Immunospot Assay (ELISpot) and Canine Macrophage Leishmanicidal Assay (CMLA)] were performed three weeks after the third vaccination (D62). The CMLA was also performed at baseline on the day of the first vaccination (D0)

LTT: This assay is designed to reveal the ability of the specific memory T cells produced as a result of vaccination to proliferate after being exposed to Soluble *Leishmania* Antigens (SLA). It was performed in a manner similar to that previously described [25], [26].

Briefly, heparinized blood samples are fractionated by centrifugation over lymphocyte separation medium. Peripheral Blood Mononuclear Cells (PBMC) obtained are incubated at a density of 10^6^ cells/ml for 5 days (37°C, 5% CO_2_) in presence of either 10 µg/ml Concanavalin A (ConA), or 10 µg/ml SLA, or with medium alone. The cells are pulsed during the last 24 h with 10 µM 5-bromo-2-deoxyuridine (BrdU), which is incorporated into the DNA of proliferating cells. BrdU incorporation is determined with a specific ELISA system (GE Healthcare, Chalfont St. Giles, UK), using peroxydase-labelled anti-BrdU antibodies which are in turn detected by a substrate reaction using 3,3′5,5′-tetramethylbenzidine. Absorbance values at 450 nm correlate directly to the amount of DNA synthesis and thereby to the number of proliferating cells in culture. The results are expressed as the lymphoproliferation index, which is the ratio of the mean optical density obtained for the SLA stimulated samples compared to the mean optical density obtained for the non-stimulated samples. ConA is used as a positive control and the medium alone is used as a negative control.

ELISpot: This assay is designed to determine the proportion of T cells that release IFN-γ after stimulation with SLA in order to quantify the level of stimulation of a specific Th1 polarity immune memory response. It was performed in a manner similar to that previously described [27]. Heparinized blood samples are fractionated by centrifugation over lymphocyte separation medium. The PBMCs obtained are incubated at a density of 10^6^ cells/ml for 3 days in multiscreen HTS filter plates (Millipore, Billerica, USA) previously coated with canine IFN-γ capture antibody (R&D System, Minneapolis, USA), in presence of 10 µg/ml ConA, or 10 µg/ml SLA antigens, or with medium alone, in a humidified 37°C CO_2_ incubator. The quantity of IFN-γ is revealed with a specific biotinylated antibody and incubation with Streptavidin-AP and the BCIP/NBT Chromogen (R&D System, Minneapolis, USA). The number of specific spots is determined by an automated ELISpot reader. ConA is used as a positive control and the medium alone is used as a negative control. The data presented are the number of spots per 2×10^5^ cells after stimulation with SLA minus the equivalent value obtained with the negative control using medium alone.

CMLA: This assay is designed to determine the ability of monocyte-derived canine macrophages to kill *Leishmania* parasites in a co-culture system due to the stimulation of iNOS expression and the resulting production of NO derivatives when the macrophage is exposed to autologous lymphocytes derived from canine PBMC. It was performed in a manner similar to that previously described [28]–[30].

Briefly, monocytes separated from lymphocytes by adherence, are cultured at a density of 2×10^5^ cells per well at 37°C and 5% CO2 for 6 days in complete RPMI 1640 medium containing 25 mM Hepes.

After 6 days of culture, monocyte-derived macrophages are infected with stationary growth phase *Leishmania infantum* (MCAN/82/GR/LEM 497) promastigotes at a ratio of 1∶5 for 5 h; then the cells are washed and fresh medium is added for 24 h and this point considered as time zero. The infected cells (t0) are washed and incubated alone or in the presence of 10^5^ autologous lymphocytes for 72 h in complete medium containing additionally 10 mM HEPES and 5×10^−5^ M 2-mercaptoethanol. After 72 h of co-culture, the lymphocytes are then removed by several gentle washings, the cell free supernatants are conserved for analysis and the macrophages are fixed in order to evaluate the leishmanial killing. One part of the fixed macrophages is stained with Giemsa and the leishmanicidal activity is determined microscopically by counting in triplicate the number of intact parasites per 100 cells with or without lymphocytes (inhibition of parasitic index).

The other part of the fixed macrophages is used to evaluate the inducible nitric oxide synthase (iNOS) expression by immunolabelling with NOS specific antibodies, as described previously [29].

The production of NO_2_ (involved in the NO cascade) is determined in the culture supernatants using the modified Griess reference technique [31]. When evaluating this leishmanicidal activity test, a result is considered as successful, when the % inhibition of the parasitic index (CMLA) is associated with the activation of the NO pathway and directly correlated with a significant increase of iNOS expression and the production of NO derivatives.

### Statistical Analyses

All statistical tests were performed using the SAS v9.1 software, and for all analyses the significance threshold was set at p = 0.05.

Intergroup comparisons of the results of the CMLA on D0 and the CMLA, LTT and ELISpot assays on D62 were performed using a Wilcoxon test.

## Results

### Serology Testing of the Humoral Immune Response

**Figure 1:**
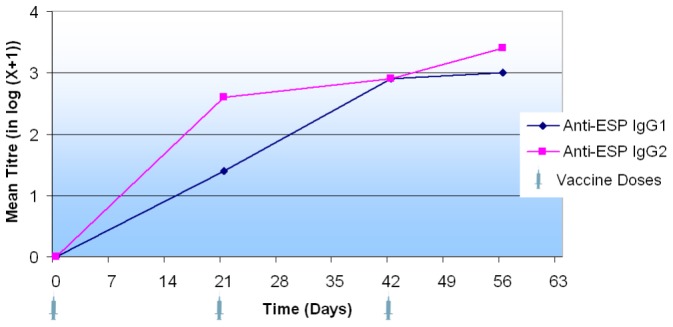
Progression in log-transformed anti-ESP IgG1 and IgG2 titres during the onset of immunity. The data presented here are the means of the log-transformed titres. The titre is taken to be the first dilution with an optical density of less than 0.4 measured at 405 nm. The sera were tested using an ESP-coated ELISA at days 0, 21, 42 and 56. The ESP used was identical in profile to the antigen of the vaccine.

**Figure 2:**
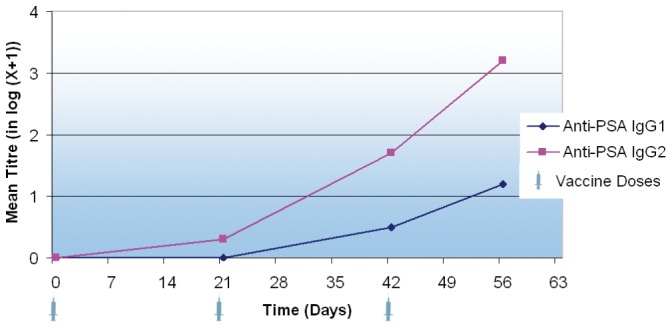
Progression in log-transformed anti-PSA IgG1 and IgG2 titres during the onset of immunity. The data presented here are the means of the log-transformed titres. The titre is taken to be the first dilution with an optical density of less than 0.4 measured at 405 nm. The sera were tested using a PSA-coated ELISA at days 0, 21, 42 and 56. PSA is a dominant antigen in ESP and therefore a key antigen in the vaccine.

Over the course of the study, all LiESP/QA-21 vaccinated dogs developed an IgG2 response to both ESP (range 1/1350 to 1/4050) and, in particular, to PSA (range 1/450 to 1/4050). See Figures 1 and 2.

All vaccinated dogs also developed an IgG1 response to ESP by the end of the study (range 1/450 to 1/4050) whereas only four of the ten vaccinated dogs developed positive IgG1 titres to PSA by day 56 (range 1/450 to 1/1350). See Figures 1 and 2. The serological response induced as a result of vaccination with LiESP/QA-21 is therefore biased towards an IgG2 profile.

No dogs in the control group developed positive titres at any point throughout the course of this study.

### Cellular Immune Response Assays

#### LTT

The cells of all animals in both vaccinated and control groups were able to respond effectively to the non-specific positive control stimulation with ConA (data not shown).

When SLA was used to determine the *Leishmania*-specific lymphoproliferation index, the median index in the control group was 1, equivalent to background cell turnover, indicating that no specific response was present.

**Figure 3:**
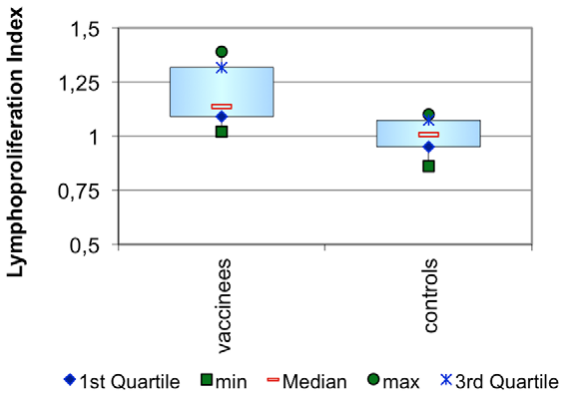
Lymphoproliferation index 3 weeks after the completion of the primary vaccination course. This assay detects the ability of the specific T cells produced as a result of vaccination to proliferate after being exposed to Soluble *Leishmania* Antigens (SLA). The lymphoproliferation index is the ratio of the mean optical density obtained for the SLA stimulated samples compared to the mean optical density obtained for the non-stimulated samples using a BrdU specific ELISA system.

In the vaccinated group, the median index was 1.2. This is significantly different from the control group (p = 0.0072) confirming the development of a specific T cell response to *L. infantum* parasites as a result of vaccination with the LiESP/QA-21 vaccine. See Figure 3.

#### ELISpot

The cells of each animal, in both groups, produced IFN-γ after stimulation with ConA (data not shown).

Once again, the control group did not show any specific response to stimulation with SLA (mean number of spots per 2×10^5^ cells was zero).

**Figure 4:**
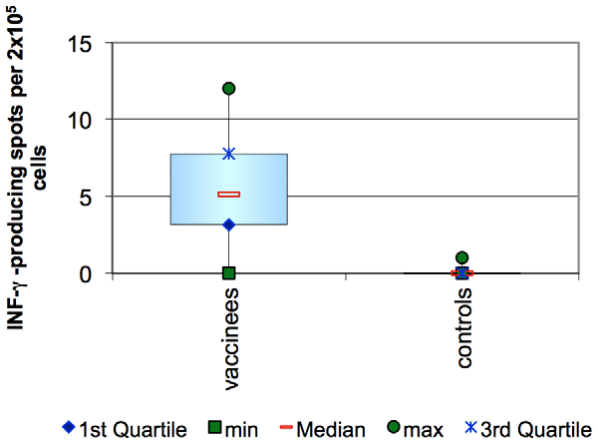
ELISpot detection of IFN-γ secreting lymphocytes 3 weeks after the completion of the primary course. This assay detects the ability of lymphocytes to secrete IFN-γ after specific stimulation with SLA by detecting spots (which represent a clone of cells secreting IFN-γ) using specific biotinylated antibodies and an automated ELISpot reader. The data presented here are the number of spots per 2×10^5^ cells after stimulation with SLA minus the equivalent value obtained with the negative control using medium alone.

In the vaccinated group the mean number of spots per 2×10^5^ cells was five, demonstrating the ability to generate IFN-γ producing T cells specific to *L. infantum* parasites as a result of vaccination with the LiESP/QA-21 vaccine. This is also significantly different from the control group (p = 0.0021). See Figure 4.

#### CMLA

Data obtained from these leishmanicidal experiments demonstrate that after LiESP/QA-21 immunization, the antileishmanial effect obtained is associated with a significant NO_2_ generation and positive iNOS expression by macrophages when compared to the NO_2_ production by control macrophages.

At baseline (day 0) the assay did not detect any significant activity in any of the dogs in either vaccinated or control groups, with all values in the three parameters being low for every dog.

**Figure 5:**
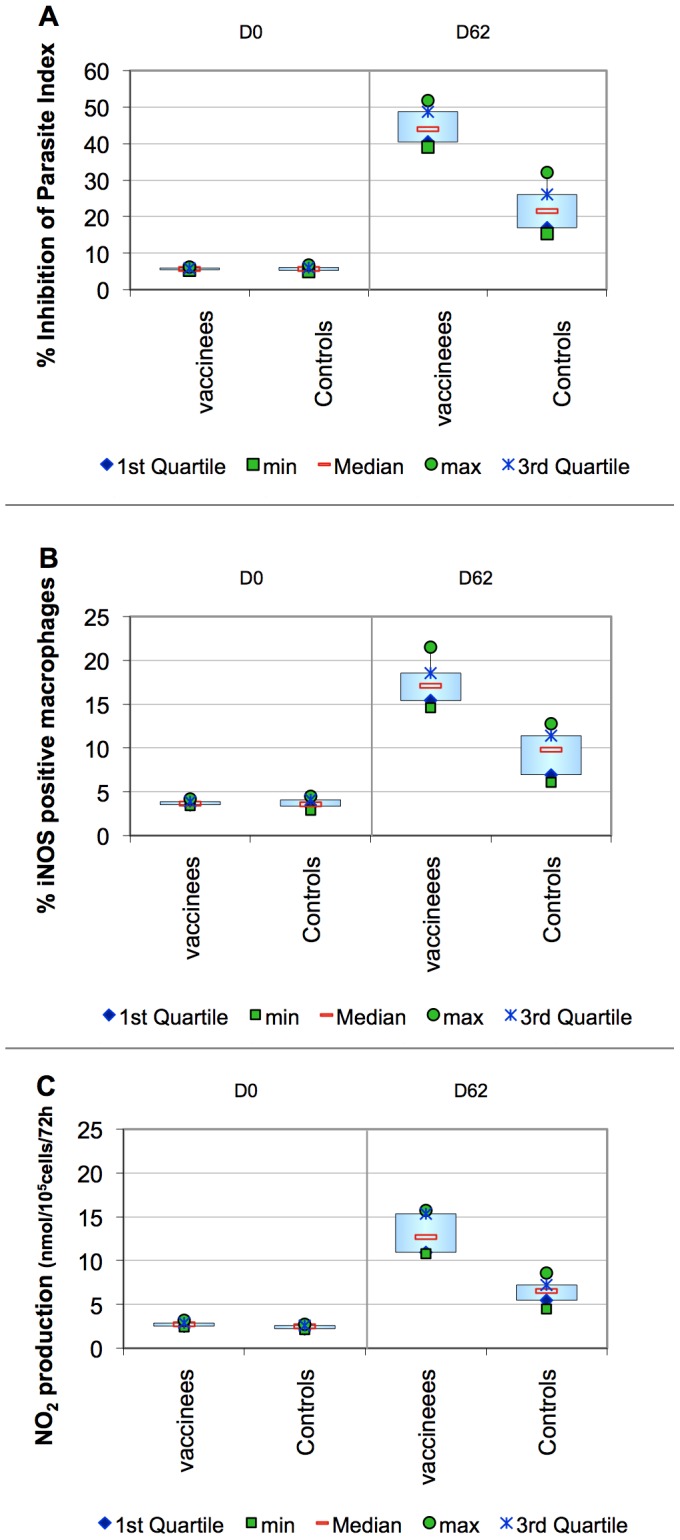
CMLA assay: inhibition of the macrophage parasitic index, iNOS activity and production of NO derivatives. Panel A is a comparison of the ability of the macrophages to inhibit parasite multiplication before vaccination (D0) and 3 weeks after completion of the primary course (D62). It demonstrates the increase in the inhibition of the macrophage parasite index as a result of vaccination. Panel B Is a comparison of the rate of expression of iNOS in the macrophages before vaccination (D0) and 3 weeks after completion of the primary course (D62). Panel C is a comparison of the rate of production of NO derivatives from the macrophages to before vaccination (D0) and 3 weeks after completion of the primary course (D62). When these three measurements are consistent, this provides evidence of an increased NO-mediated pathway of parasite killing as a result of vaccination.

There was no statistical difference between the vaccinated and control groups at baseline for the CMLA index (p = 0.9701) and iNOS activity (p = 0.6002). However, the NO_2_ production was higher in the vaccinated group than in the control group (p = 0.0335). This was then taken into account for the analysis of the data on day 62 by performing an additional analysis of co-variance. (3 weeks after the third dose of the vaccine). See Figure 5.

On day 62, the vaccinated group had significantly higher results than the control group in all three parameters (p = 0.0014). Taking into account the heterogeneity in the values from D0, the NO_2_ production was still significantly higher at D62 in the vaccinated group in comparison to that seen in the control group (p<0.0001).

In terms of temporal progression, both groups demonstrated a rise in the values measured by these tests. However only the vaccinated dogs achieved values that were above the threshold in all three tests (See figure 5).

It is evident from these results that successful vaccination with the LiESP/QA-21 vaccine induces protective antigen-activated T cells producing Th1-derived cytokines such as IFN-γ. These cells are capable of activating autologous infected macrophages to kill intracellular *L. infantum* parasites by means of induction of iNOS and the production of nitric oxide (NO) derivatives.

## Discussion

Resistance to canine leishmaniasis is associated with a predominance of Th1 cytokines, such as IFN-γ, favouring a Th1 bias in an overall mixed Th1/Th2 response [18], [32]. By contrast, progression to disease, and potentially death, is associated with a predominance of Th2 cytokines and a marked humoral response in the absence of a strong Th1 response [19].

In mice it has been shown that antibodies of the IgG1 subtype are associated with a Th2 response and antibodies of the IgG2 subtype are associated with a Th1 response [33]. However, despite some initial reports suggesting the same happens in dogs, the IgG1/IgG2 ratio does not appear to be correlated with resistance to development of the disease [34].

Notwithstanding the recent evidence refuting the direct relationship between the IgG1/IgG2 ratio and Th1/Th2 balance, it is still possible that the IgG2 response to PSA could ultimately prove relevant to some degree. IgG2a antibody, which is effective in opsonisation and complement fixation, may still have a direct role by targeting individual amastigotes that are released from infected macrophages [34]. A recent investigation looked at the activity of sera from dogs vaccinated with a prototype LiESP vaccine formulated with the MDP adjuvant [35]. It found that the IgG2 induced by LiESP vaccination resulted in a functionally active serum that was leishmanicidal to both promastigotes and amastigotes, that had a strong inhibitory effect on the *in vitro* growth of both stages and, importantly, that pre-treatment of amastigotes by the serum led to a significant inhibition of *in vitro* infectivity to canine macrophages. The authors concluded that even if the Th1-dominated cell-mediated immune response is the primary mechanism of resistance, it is possible that IgG2 may play a role in the complex overall immune responses that lead to resistance in the dog. It is therefore of interest to note that in the present study we had a clear bias towards IgG2 production and that this was most obvious in the response against PSA. The PSA of *L. infantum* has been specifically demonstrated to have a role in macrophage invasion [36], and this fact, taken in association with the findings of Bourdoiseau *et al*, [35] suggests that the ELISA results presented here should not be discounted. Further work to explore this in more detail could be very interesting, as would be an examination of the antibody response profile to vaccination with LiESP/QA-21 using the newer canine IgG1-4 monoclonal antibodies, even if these have not yet been correlated to a specific Th1/Th2 profile [34].

It should also be noted that even if raised titres to ESP are achieved after 2 injections, the elevated IgG2 titres to PSA are obtained only after the third injection. This is due to a combination of dogs which had only low titres after the second injection displaying augmented titres after the third, and some dogs which were still negative on this test after the second injection displaying detectable titres for the first time after the third.

The data presented in this study regarding cellular immunity parameters are particularly interesting and, according to the current state of knowledge of the immunology of this disease, they are the more applicable results. Although there appears to be a clear consensus that the Th1-dominated profile within a mixed Th1/Th2 response is the desirable profile for protection of the dog [37], the overall *in vivo* response is clearly complex and it is not possible to correlate individual markers of this response with absolute resistance to disease in a particular dog. Therefore it is important to take all of the parameters together to obtain a sense of the overall direction of the response. This concept was clearly presented in a recent paper by Reis *et.al.*, where the concept of a dynamic spectrum in the immune response was introduced [11]. In the present study it is clear that use of the LiESP/QA-21 vaccine induces a subset of *Leishmania*-specific T cells as evidenced by the specific proliferative response upon stimulation with crude extracts of the parasite. We also showed that this specific T cell population has a dominant Th1 profile as evidenced by the ability to secrete IFN-γ upon stimulation with SLA. Although there is no single cell-mediated immunity marker that is directly correlated with protection in individual patients, much data has accumulated to support IFN-γ production from stimulated PBMCs as a key requirement. In a study of the expression of 6 cytokine markers (IFN-γ, TNF-α, IL-2, IL-4, IL-5 and IL-10) in the memory T-cell response to SLA stimulation in humans, only IFN-γ production was correlated with cured/resistant patients [38]. Further studies have confirmed that this is also applicable to dogs, since the ability of canine PBMCs to proliferate and produce IFN-γ at high levels after stimulation with SLA was associated with a resistant profile in contrast to polysymptomatic and non-infected control dogs [39], [40].

Care must be taken in the over-interpretation of a single parameter such as IFN-γ as we know that it operates as part of a complicated network of regulatory and counter-regulatory interactions involving multiple cytokines [41]. Nonetheless, the CMLA results presented here further demonstrate that the IFN-γ production was functionally active *in vitro* where it stimulated autologous macrophages to kill *L. infantum* parasites. This finding is important, as it appears that although IFN-γ production from stimulated PBMCs is the key marker of the correct response, other co-factors such as TNF-α are also needed to effect the stimulation that results in effective leishmanicidal activity in the macrophage [19]. In murine and human macrophages, TNF-α and IFN-γ act synergistically to induce the elimination of *Leishmania* amastigotes [42], [43], and this has been confirmed to be the same in dogs also [11]. Recent studies have also demonstrated that in the presence of high levels of IL-10, even significant IFN-γ responses may not be effective and the IL-10/IFN-γ ratio could be relevant [41]. Unfortunately due to the lack of an available validated assay for canine IL-10 this was not possible during this study.

The data presented here are consistent with an overall effective response. Furthermore, when the CMLA results are considered there is a consistent clear correlation between induction of iNOS, production of NO derivatives and leishmanicidal effect. This suggests that iNOS positive activated macrophages were able to control the multiplication of *Leishmania* parasites and to kill them. This is consistent with the current views on the mechanism of parasite killing or parasite maintenance in the macrophage, whereby stimulation of macrophages by Th1 cytokines favours iNOS metabolism of L-arginine to leishmanicidal NO [44], but stimulation by Th2 cytokines leads to an alternative activation pathway in the macrophages. This favours arginase metabolism of L-arginine, resulting in the synthesis of polyamines which sustain the growth of the intracellular parasite burden [45]. The critical role played by induction of NO production and the oxidative burst in killing *Leishmania* parasites has been well documented in recent years and was elegantly demonstrated in a Syrian hamster model which showed that the inability to produce NO due to a lack of iNOS expression resulted in the inability to control intracellular *L. donovani* [46]. The pivotal role of NO production in dog macrophages had also been demonstrated in a previous study showing that after successful chemotherapy the macrophage regained the ability to control the parasites via an IFN-γ mediated stimulation of the NO synthase pathway [30]. In light of this, it could also have been interesting to have looked for evidence of alternatively activated macrophages which are tolerant to the parasite, or to have investigated the production of Th2 cytokines, such as IL-4 and IL-10, that are able to down-regulate the antileishmanial effect of macrophages by decreasing NO production [47].

It is also interesting to note the rise in the CMLA results in the control group during the study. This is not entirely surprising, as it is well recognised that the maturation of the cell-mediated arm of the adaptive immune response is slower than that of the humoral response ability [48], and this increase may simply reflect progressive maturity of the immune response in these dogs between the ages of 6 and 9 months. Nevertheless, despite the small rise in the control group, the clear difference between the groups over the course of this study as a result of the application of the LiESP/QA-21 vaccine adds another piece of evidence supporting the expected efficacy of the vaccine in dogs.

It must also be noted that such data cannot represent the full picture of the complex immune response to *L. infantum*. Other factors are also in play in the context of the intact immune system of a live animal. This means that such data can never completely replace *in vivo* challenge studies. However, in the context of attempts to reduce the use of experimental animals in virulent challenge studies, models such as this one can provide a very valuable database and they have been recommended as a rational way to explore the activity of any potential vaccine against canine leishmaniasis [11]. Despite this obvious limitation, the overall result here clearly demonstrates that the presence of sensitized lymphocytes, induced as a result of vaccination with LiESP/QA-21, enhanced the antileishmanial activity of autologous macrophages and enabled them to kill *L.infantum* parasites *in vitro*, via the nitric oxide pathway. This supports the hypothesis that this vaccine could be expected to be efficacious at reducing parasite loads *in vivo*.

### Conclusion

The results presented in this study confirm that vaccination of dogs with LiESP/QA-21 vaccine is capable of inducing a Th1 profile cellular response, within 3 weeks of the primary vaccine course, which in turn is effective *in vitro* at reducing the parasite load in pre-infected autologous macrophages. Until *in vivo* challenge studies are reported, this data provides an understanding of the mechanism and onset of immunity induced by use of LiESP/QA-21 vaccine, which is the first of its type commercially available in Europe.
